# Metabolite profiling and transcriptome analyses reveal novel regulatory mechanisms of melatonin biosynthesis in hickory

**DOI:** 10.1038/s41438-021-00631-x

**Published:** 2021-09-01

**Authors:** Wenchao Chen, Jiaqi Zhang, Shan Zheng, Zhanqi Wang, Chuanmei Xu, Qixiang Zhang, Jiasheng Wu, Heqiang Lou

**Affiliations:** 1 State Key Laboratory of Subtropical Silviculture, Zhejiang A&F University, 311300 Hangzhou, Zhejiang China; 2 Key Laboratory of Vector Biology and Pathogen Control of Zhejiang Province, College of Life Sciences, Huzhou University, 313000 Huzhou, China

**Keywords:** Gene regulation, Plant hormones

## Abstract

Studies have shown that melatonin regulates the expression of various elements in the biosynthesis and catabolism of plant hormones. In contrast, the effects of these different plant hormones on the biosynthesis and metabolism of melatonin and their underlying molecular mechanisms are still unclear. In this study, the melatonin biosynthesis pathway was proposed from constructed metabolomic and transcriptomic libraries from hickory (*Carya cathayensis* Sarg.) nuts. The candidate pathway genes were further identified by phylogenetic analysis, amino-acid sequence alignment, and subcellular localization. Notably, most of the transcription factor-related genes coexpressed with melatonin pathway genes were hormone-responsive genes. Furthermore, dual-luciferase and yeast one‐hybrid assays revealed that CcEIN3 (response to ethylene) and CcAZF2 (response to abscisic acid) could activate melatonin biosynthesis pathway genes, a tryptophan decarboxylase coding gene (*CcTDC1*) and an *N*-acetylserotonin methyltransferase coding gene (*CcASMT1*), by directly binding to their promoters, respectively. Our results provide a molecular basis for the characterization of novel melatonin biosynthesis regulatory mechanisms and demonstrate for the first time that abscisic acid and ethylene can regulate melatonin biosynthesis.

## Introduction

As an important neurohormone for mammals, melatonin is involved in many biological processes in animals^1^. Melatonin was first found in the pineal gland of cows in 1958^2^. In 1995, melatonin was initially identified in plants^3^. Since then, the biological functions of melatonin have attracted extensive attention from plant scientists. As a biostimulator in plants, melatonin has also been considered to promote the growth and development of plants, including coleoptile growth, root growth, leaf morphology, flowering time, and fruit ripening^4,5^. Furthermore, melatonin also acts as a signaling molecule, mediating the plant defense response to pathogen attacks through the mitogen-activated protein kinase (MAPK) pathway^6–8^. Large numbers of experiments have confirmed that melatonin also plays vital roles in resisting various abiotic stresses, including oxidative^9^, heavy metal^10^, high temperature^11^, cold^12^, senescence^13^, drought^14^, aluminum^15^, and salt stresses^16^. Notably, melatonin can improve sleep quality and has been utilized by humans to overcome jetlag^17^. Moreover, melatonin has also been utilized by humans as a dietary supplement due to its perceived antioxidant activity^18^. These essential characteristics have prompted scientists to continue to study the biological functions, biosynthetic pathways, and regulation of melatonin in plants.

There are four enzymatic reactions in melatonin biosynthesis from tryptophan. At present, at least six enzymes are known to be involved in this process, so there are at least four biosynthetic pathways for melatonin^19^. The order of enzyme reactions in the melatonin biosynthetic pathway not only changes the type and synthesis sites of intermediates but also affects the formation of melatonin. The first step of melatonin synthesis is the catalysis of tryptophan to form tryptamine by tryptophan decarboxylase (TDC) in the cytoplasm, following which tryptamine is converted to serotonin by tryptamine 5-hydroxylase (T5H) in the endoplasmic reticulum (ER). However, there is also a reverse step where hydroxylation occurs first in the cytoplasm, although putative tryptophan hydroxylase (TPH) has not been identified in plants^19,20^, and 5-hydroxytryptophan is decarboxylated to serotonin by TDC in the cytoplasm^21,22^. Serotonin can be catalyzed by serotonin *N*-acetyltransferase (SNAT) to produce *N*-acetylserotonin in chloroplasts and then by *N*-acetylserotonin methyltransferase (ASMT)/caffeic acid *O*-methyltransferase (COMT) to produce melatonin in the cytoplasm. An alternative pathway for melatonin biosynthesis is also present, in which serotonin could be methylated into 5-methoxytryptamine by ASMT (or together with COMT) in the cytoplasm, and then 5-methoxytryptamine can be acetylated by SNAT to produce melatonin in the chloroplast.

It has been proven that melatonin affects the biosynthesis and catabolism of auxin, gibberellin, cytokinin, abscisic acid (ABA), ethylene, jasmonic acid, salicylic acid, and brassinosteroids by regulating the expression of pathway-related enzymes, receptors, and transcription factors^23^. The application of exogenous melatonin increased IAA contents by upregulating the expression of *IAA19*, *IAA24*, and *PIN* in tomato seedlings^24^ and the expression of IAA-amino synthase genes and *YUCCAs* in Arabidopsis seedlings and roots^25,26^. Similar findings were also confirmed in other species, such as *Brassica juncea*^27^. In addition, melatonin can also affect the synthesis and metabolism of GAs through increased expression of *GA20ox* and *GA3ox* in cucumber seedlings under saline conditions^28,29^. Moreover, under salt and drought stress, melatonin upregulated the expression of ABA catabolism genes (*CYP707A1* and *CYP707A2*) and downregulated the expression of a key enzyme (NCED) of ABA biosynthesis, thus decreasing the content of ABA^28,30^. In addition, melatonin pretreatment of watermelon plants at cold temperatures can also downregulate the expression of the ABA receptor gene *PYL8*^31^. Zhang et al. found that two cytokinin biosynthesis genes, *IPT2* and *OG1*, were upregulated by melatonin under stress conditions, which resulted in an increase in cytokinin^32^. In addition, melatonin also affects the biosynthesis of ethylene and brassinosteroids by regulating ethylene pathway genes and brassinosteroid regulators, respectively^25,33–35^. This evidence suggests that exogenous melatonin can change endogenous plant hormone contents by changing the expression of the corresponding synthesis genes, receptors, and transcription factors. However, it has not been proven that these different plant hormones can affect the melatonin levels of plants.

In addition, the regulators of melatonin synthesis pathway genes remain obscure. Recently, two transcription factors*, MeRAV1* and *MeRAV2*, were shown to regulate *MeTDC2*, *MeT5H*, and *MeASMT1* directly and to indirectly regulate other melatonin biosynthesis genes (*MeTDC1*, *MeASMT2*, *MeASMT3*, and *MeSNAT*) in cassava^36^. Wei et al. reported that two transcription factors, MeWRKY79 and MeHsf20, could activate the transcription of *MeASMT2* and melatonin biosynthesis in cassava^37^. Hsf1a can bind to the promoter of *COMT1*, leading to its upregulation and melatonin accumulation^38^. In cassava, MeWRKY20/75, as the common interacting proteins of MeTDC2, MeASMT2, and MeASMT3, can positively regulate melatonin accumulation^39^.

Hickory (*Carya cathayensis* Sarg.) belongs to the walnut family Juglandaceae and is native to eastern China, where it has been cultivated for consumption for more than 500 years since the Ming Dynasty. It is mainly distributed in the Tianmu Mountain area in southern Anhui Province and northeast Zhejiang Province. The kernel of hickory is a good tonic and has been claimed to have multiple biological functions, such as reducing the incidence of cancer, atherosclerosis and cardiovascular disease, and benefits for consenescence and sex capacity, but there is no epidemiological evidence of the latter claims. As mentioned above, melatonin is a bioactive component with a variety of biological functions. However, the content of melatonin and its biosynthesis pathway as well as the underlying molecular regulatory mechanisms of melatonin accumulation in hickory are still unclear.

Therefore, we employed transcriptome and metabolome profiling of embryos at different developmental stages to identify the biosynthetic pathways of melatonin. We further identified candidate genes that are potentially involved in the biosynthesis of melatonin in hickory based on phylogenetic and amino-acid sequence alignment analyses. Moreover, we identified the subcellular localization of melatonin biosynthetic pathway enzymes to clarify where they function. Pearson correlation analysis was performed to identify transcription factors coexpressed with melatonin pathway genes. Surprisingly, we found that most of the identified transcription factors are hormone-responsive genes. The results of a dual-luciferase assay showed that EIN3 and AZF2 could upregulate the expression of CcTDC1 and ASMT1, respectively, suggesting that these transcription factors are involved in the regulation of melatonin biosynthesis. Overall, our study identified the melatonin biosynthesis pathway and candidate genes encoding enzymes involved in the biosynthesis of melatonin and proved that other plant hormones may regulate melatonin biosynthesis in hickory.

## Materials and methods

### Plant materials

During the ripening stage from mid-August to mid-September, hickory fruits were collected from the orchard of Zhejiang A&F University in Hangzhou, China. After collection, the pericarp and testa of fruits were removed immediately, and the remaining embryos were quickly frozen in liquid nitrogen and then stored at −80 °C. From the collected samples, we selected nine embryos with different degrees of maturity for further analysis (named CTB1, CTB2, CTB3, CTC1, CTC2, CTC3, CTD1, CTD2, and CTD3). The embryos of the CTB, CTC, and CTD groups were collected on August 15, August 30, and September 15, 2018, respectively. Each embryo was ground to a powder in liquid nitrogen and divided into two parts: one was used for RNA extraction, and the other was used for liquid chromatography-tandem mass spectrometry (LC-MS/MS**)** analysis.

### Metabolite analysis by LC-MS/MS

Extraction and analysis of metabolites were carried out by Metware Biotechnology Co. Ltd. (Wuhan, China). The freeze-dried embryos were ground to a power and extracted by 70% aqueous methanol. After centrifugation at 10,000 × *g* for 10 min, all supernatants were combined and filtered through a 0.22 mm pore size membrane and then analyzed by an LC-ESI-MS/MS system (UPLC, Shim-pack UFLC SHIMADZU CBM30A system, www.shimadzu.com.cn/; MS/MS, Applied Biosystems 6500 Q TRAP, www.appliedbiosystems.com.cn/). The effluent was alternatively connected to an ESI-triple quadrupole-linear ion trap (Q TRAP)-MS. Linear ion trap (LIT) and triple quadrupole (QQQ) scans were acquired on a triple quadrupole-linear ion trap mass spectrometer equipped with an ESI Turbo Ion-Spray interface, operating in positive ion mode and controlled by Analyst 1.6.3 software (AB Sciex). A scheduled multiple reaction monitoring method was used to quantify metabolites. To generate the maximal signal, the collision energy and declustering potential were optimized for each precursor-product ion (Q1-Q3) transition^40^. The melatonin content was calculated from the quantitative data of melatonin obtained above and the standard curves acquired from an authentic melatonin standard.

### RNA extraction and RNA-Seq

Total RNA of the samples was extracted with an RNAprep Pure Plant kit (DP441, Tiangen, China). Illumina RNA-Seq was performed by Metware Biotechnology Co. Ltd. (Wuhan, China). The RNA quality was detected by a NanoPhotometer spectrophotometer (IMPLEN, CA, USA), Qubit 2.0 Fluorometer (Life Technologies, CA, USA), and Agilent Bioanalyzer 2100 system (Agilent Technologies, CA, USA). The poly(A) mRNA was enriched by magnetic beads with oligo (dT). The mRNA was randomly fragmented. First-strand cDNA was synthesized using the M-MuLV reverse transcriptase system. The RNA strand was then degraded by RNase H, and second-strand cDNA was synthesized using DNA polymerase. The double-stranded cDNAs were ligated to sequencing adapters. The cDNAs (~200 bp) were screened using AMPure XP beads. After amplification and purification, cDNA libraries were obtained and sequenced using the Illumina HiSeqTM 2000 system.

### Sequence data processing

The raw reads were transformed from the sequencing raw image data by CASAVA base recognition. To obtain high-quality data, adapters of sequences were cut, and low-quality reads with ≥5 uncertain bases or with over 50% Qphred ≤20 bases were removed using fastp^41^. The GC-content of clean reads was calculated. The Q20 and Q30 values were also produced by FastQC to evaluate the base quality.

Then, the clean reads were mapped to the hickory reference genome using HISAT with default parameters^42,43^. Gene expression levels were determined using the RPKM (reads per kb per million reads) method^44^.

### Real-time RT-PCR analysis

Purified RNA (1 µg for each sample) was reverse transcribed to first-strand cDNA with a cDNA Reverse Transcription Kit (PrimeScript^TM^ RT Master Mix, Takara) based on the manufacturer’s instructions. The primers are listed in Supplementary Table S1. qRT-PCR was conducted with a ChamQ SYBR qPCR Master Mix kit (Vazyme) and a C1000 Touch™ Thermal Cycler system (Bio-Rad). Relative transcript levels were calculated according to the 2^−ΔΔCp^ method using a housekeeping gene, *CcActin*, for reference. Three biological and technical replications were performed.

### Transient fluorescent protein expression assay in *Nicotiana benthamiana* leaves

Genes used for investigation of subcellular localization were amplified from cDNA using PrimeSTAR^®^ HS (Premix, Takara) and introduced into the *35* *S::GFP* vector (modified from pCAMBIA1300) using a ClonExpress^®^II One Step Cloning Kit (Vazyme, China). The primers are specified in Supplementary Table S1. All constructs were introduced into *Agrobacterium tumefaciens* strain GV3101. Positive clones were grown in LB medium with kanamycin at 28 °C until the OD_600_ reached 0.5. After centrifugation at 6000 rpm for 5 min at 4 °C, the supernatants were removed, and the agrobacteria were resuspended using infiltration medium containing 10 mM MgCl_2_, 0.2 mM acetosyringone, and 10 mM MES (pH 5.6) and brought to an OD_600_ of 0.5–1.0. Before being infiltrated, agrobacteria carrying constructs with the GFP signal and marker vectors with the RFP signal were mixed in a 1:1 ratio. For transient expression of the fluorescent proteins, infiltration buffer was injected into leaves of *Nicotiana benthamiana*. After 3 days of incubation, fluorescent signals were detected by confocal laser scanning microscopy (LSM510, Karl Zeiss) at room temperature.

### Plant hormone treatment

Ethrel (500, 1000, or 1500 mg/L) and ABA (50, 100, or 150 mg/L) were sprayed once a day for 7 days, from July 24 to July 30, onto the pericarps of hickory grown in the orchard of Zhejiang A&F University. Spray application of water was used as a control.

### Dual-luciferase assay

To determine the transactivation activity of transcription factors to the promoters of coexpressed melatonin biosynthesis genes, a transient dual-luciferase assay was performed. The coding regions of transcription factors were cloned into the pCAMBIA1300 vector under the control of the CaMV35S promoter as an effector. The fragments of the promoters of melatonin biosynthesis genes were introduced into the pGreenII 0800-LUC vector, allowing the promoter fragments to be cloned as a transcriptional fusion with the firefly luciferase gene (LUC). The constructed effector and reporter plasmids were introduced into *Agrobacterium tumefaciens* (GV3101) and then cotransformed into tobacco. All primers are listed in Supplementary Table S1. LUC and REN luciferase activities were measured using a dual-luciferase assay kit (Promega) and a Luminoskan Ascent Microplate Luminometer (Thermo Fisher Scientific). The results were calculated by the ratio of LUC to REN.

### Yeast one-hybrid assay

The yeast one-hybrid assay was performed using the MATCH-MAKER Gold Yeast One-Hybrid Library Screening System (Clontech). The ORFs of transcription factors were cloned in frame after the transcriptional activation domain of yeast GAL4 in pGADT7. The promoter fragments of *CcTDC1* and *CcASMT1* were cloned upstream of the aureobasidin A (AbA) resistance reporter gene (AUR1-C) in the pAbAi vector. Primers are listed in Supplementary Table S1. Pairs of plasmids were introduced into yeast strain Y1H Gold and cultured on SD medium without Leu containing 0–300 ng/ml AbA at 30 °C for 72 h.

### Statistical and sequence analyses

Correlations among data were calculated by Pearson’s correlation coefficients (r) using SPSS, version 16.0 (SPSS, Inc., Chicago, IL, U.S.A.). Significant differences were determined using Duncan’s new multiple range test at *p* < 0.05. Phylogenetic analysis was performed based on the deduced amino-acid sequences of melatonin biosynthesis pathway enzymes from hickory and other plants using a bootstrap neighbor-joining evolutionary tree by MEGA 7.0 software with 1000 bootstrap replicates. Amino-acid sequence alignment was conducted with DNAMAN software (version 9).

## Results

### Metabolic profiling of hickory embryos and quantitative analysis of melatonin

**Fig. 1:**
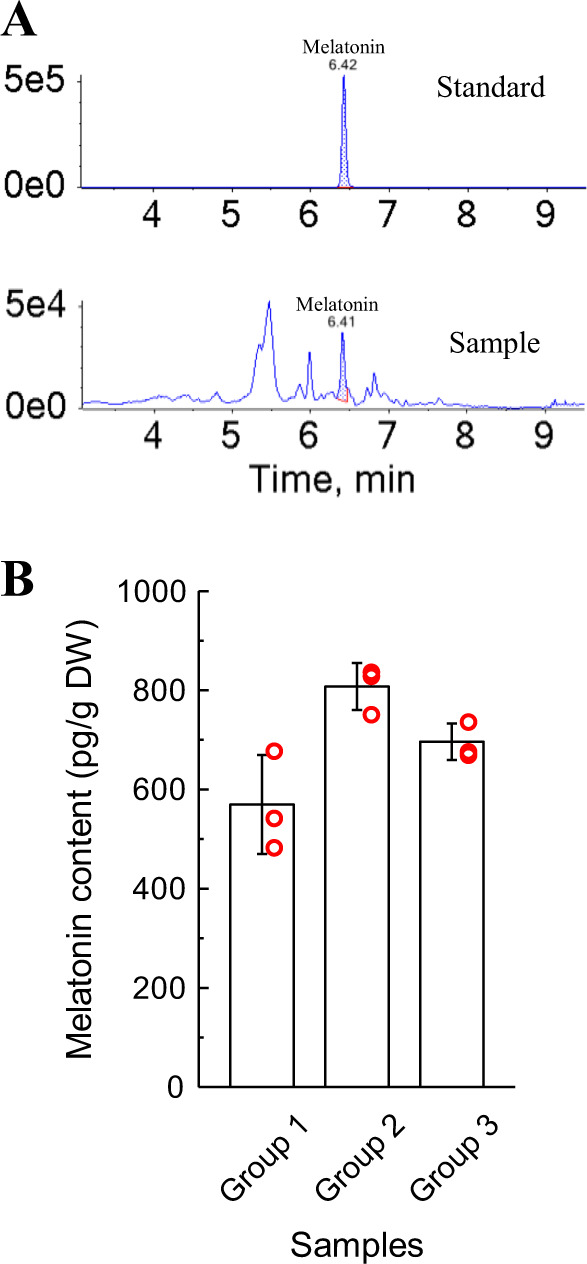
Melatonin content of hickory embryos. **A** Chromatograms of the melatonin standard and a representative sample. **B** Melatonin contents of nine embryos with different degrees of maturity (each red circle represents an embryo). The maturity increased from groups 1–3. Data are presented as the mean ± SD

To understand the melatonin level of hickory fruits and elucidate its biosynthesis pathway, we selected nine embryos in the ripening stage with different degrees of maturity for metabolite quantification using a broadly targeted LC-MS/MS-based metabolic profiling method. A total of 608 annotated metabolites were identified (Supplementary Table S2). From the database, we found that melatonin existed in hickory embryos. Furthermore, we quantified melatonin using authentic melatonin standards (Fig. 1A). The results showed that the melatonin content among the embryos of different maturities varied and was high, ranging from 485 to 839 pg/g dry weight of embryo (Fig. 1B). To investigate the synthetic pathway of melatonin, metabolites that may be intermediates for melatonin synthesis were selected and are listed in Table 1. The parameters used to identify these metabolites are also presented in Table 1. Their relative contents (represented as peak areas) are shown in Supplementary Table S2. L-tryptophan, L-tryptamine, 5-hydroxytryptophan, serotonin, *N*-acetylserotonin, and melatonin were detected. However, 5-methoxytryptamine was not detected in hickory embryos. These results suggested that COMT/ASMT may have no catalytic activity for serotonin or that SNAT has higher catalytic efficiency toward 5-methoxytryptamine in hickory, making the 5-methoxytryptamine level too low to detect.

**Table 1:** Six metabolites involved in melatonin biosynthesis in hickory embryos

Compounds	Q1 (Da)	Q3 (Da)	Rt (min)	Molecular weight (Da)	Ionization model
L-tryptophan	203.0	116.1	2.39	204.0899	[M − H]−
N-acetylserotonin	219.0	160.1	3.32	218.1060	[M + H]+
5-hydroxy-L-tryptophan	221.1	114.8	1.66	220.0850	[M + H]+
L-tryptamine	161.1	144.2	2.74	160.1000	[M + H]+
Serotonin	177.0	160.2	1.63	176.0950	[M + H]+
Melatonin	231.0	144.1	4.16	232.1210	[M − H]−

### De novo sequence assembly, functional annotation, and validation of RNA-seq data

To further elucidate the melatonin biosynthesis pathway at the transcriptional level, nine cDNA libraries, which were constructed from total RNAs, were subjected to high-throughput parallel sequencing. After removing adaptor sequences and low-quality reads, total clean reads and clean bases were generated, ranging from 45.96 to 64.39 million (M) and 6.89 to 9.66 Gb, respectively. The GC percentages ranged from 46.13 to 48.18%. The Q20 and Q30 values used to assess the quality of the sequencing bases were also obtained, ranging from 96.89 to 97.57% and 91.84 to 93.29%, respectively. The percentage of clean reads per library mapped to the hickory reference genome ranged from 95.83% to 96.94% (Supplementary Table S3).

To confirm the expression levels of genes from RNA-Seq data, the transcriptional abundance of 13 genes randomly selected from the melatonin biosynthesis pathway was detected using qRT-PCR (Supplementary Fig. S1A-M). The results showed a high correlation between the qRT-PCR and RNA-Seq data with an r of 0.720 (*p* < 0.001), suggesting that the expression data from RNA-seq were reliable (Supplementary Fig. S1N).

### Identification of the genes potentially involved in melatonin biosynthesis

**Fig. 2:**
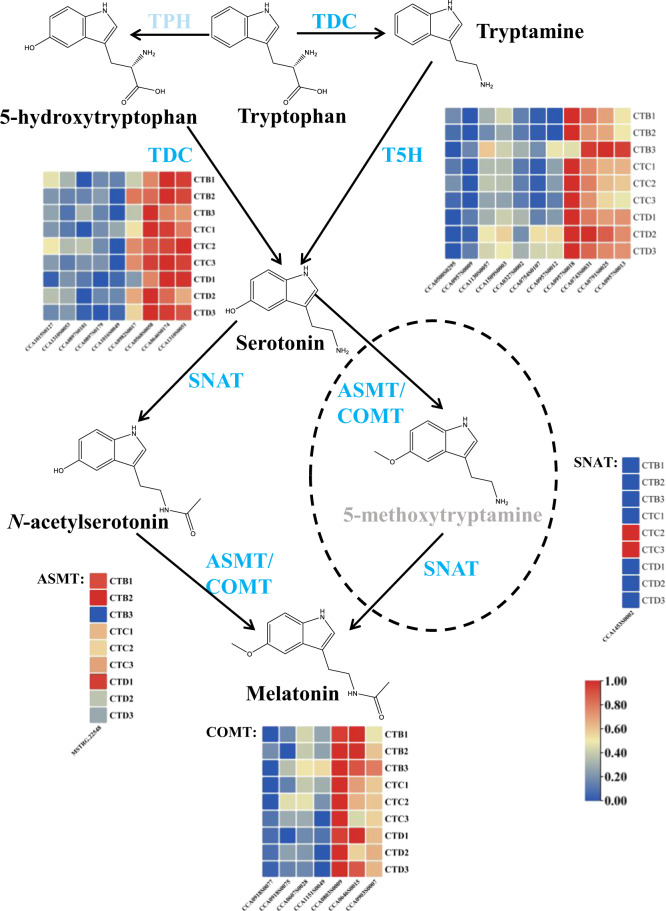
Putative pathways for melatonin biosynthesis in hickory embryos and expression levels of the candidate enzyme genes involved in melatonin biosynthesis. Light-colored words represent the genes or metabolites that were not detected in this study. The nine squares of each enzyme correspond to the nine embryos

Six enzymes were involved in four melatonin biosynthesis pathways in plants. We tried to identify all the genes annotated as encoding the six enzymes to further identify the melatonin biosynthesis pathway at the transcriptional level in hickory embryos. Interestingly, the genes encoding the enzymes TDC (9 genes), T5H (11 genes), SNAT (1 gene), COMT (7 genes), and ASMT (1 gene) were expressed in hickory embryos, while we could not find any genes encoding TPH (Fig. 2).

To identify the possible candidate genes, we evaluated the evolutionary relationships between the genes encoding proteins from hickory and known enzymes involved in melatonin synthesis from other plants. As shown in Supplementary Fig. S2, CCA0568S0058 was most closely clustered with JrTDC2, followed by CaTDC (Supplementary Fig. S2A). CCA0743S0031 was most closely clustered with JrT5H and OsT5H (Supplementary Fig. S2B). MSTRG.22548 and MSTRG.22549 were most closely clustered, and they had high homology with JrASMT (Supplementary Fig. S2C). CCA0646S0015 and CCA0903S0007 were closely clustered, and they were most closely clustered with JrCOMT, followed by MsCOMT (Supplementary Fig. S2D). CCA1453S0002 was most closely clustered with JrSNAT2 (Supplementary Fig. S2E).

**Fig. 3:**
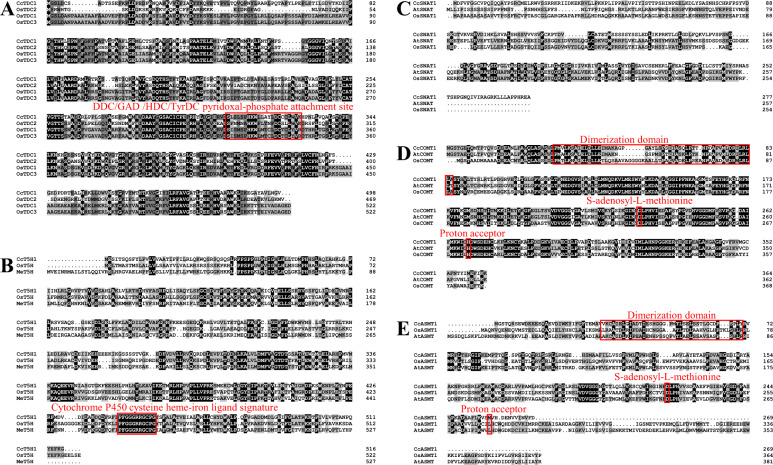
Amino-acid sequence alignment of proteins involved in melatonin biosynthesis from hickory and other plants. **A** Alignment of the deduced amino-acid sequences of CcTDC1 (CCA0568S0058) with OsTDC1 (XP_015648768.1), OsTDC2 (XP_015644906.1) and OsTDC3 (NM001067504). The conserved DDC/GAD/HDC/TyrDC pyridoxal-phosphate attachment site is indicated. **B** Alignment of the deduced amino-acid sequences of CcT5H1 (CCA0743S0031) with OsT5H (XP_015618264.1) and MeT5H (XP_021614605.1). The conserved cytochrome P450 cysteine heme-iron ligand signature is indicated. **C** Alignment of the deduced amino-acid sequences of CcSNAT1 (CCA1453S0002) with AtSNAT (At1g32070), OsSNAT1 (AK059369) and OsSNAT2 (XP_015648698.1). **D** Alignment of the deduced amino-acid sequences of CcCOMT1 (CCA0646S0015) with AtCOMT (XP_015650053.1) and OsCOMT (AK064768). The conserved dimerization domain, S-adenosyl-L-methionine, and proton acceptor are indicated. **E** Alignment of the deduced amino-acid sequences of CcASMT1 (MSTRG.22549) with AtASMT (At4g35160) and OsASMT1 (XP_015610997.1). The conserved dimerization domain, S-adenosyl-L-methionine, and a proton acceptor are indicated

CCA0568S0058 (named CcTDC1), CCA0743S0031 (named CcT5H1), MSTRG.22548 (named CcASMT1), CCA0646S0015 (named CcCOMT1), and CCA1453S0002 (named CcSNAT1) were selected as candidate transcripts of enzymes in the melatonin biosynthesis pathway based on phylogenetic analysis. The protein domains and functional sites of each candidate gene encoding an enzyme were predicted by PROSITE (https://prosite.expasy.org/prosite.html). The CcTDC1 protein sequence contained a DDC/GAD/HDC/TyrDC pyridoxal-phosphate attachment site that was similar to those of OsTDC1, OsTDC2, and OsTDC3 (Fig. 3A). The CcT5H1 protein sequence contained a highly conserved cytochrome P450 cysteine heme-iron ligand signature along with other known T5Hs (Fig. 3B). The CcSNAT1 protein sequence was similar to the OsSNAT1 and AtSNAT protein sequences (Fig. 3C). A dimerization domain, S-adenosyl-L-methionine, and proton acceptor were predicted in both the CcASMT1 and CcCOMT1 protein sequences (Fig. 3D and E).

### Subcellular localization of candidate transcripts encoding enzymes

**Fig. 4:**
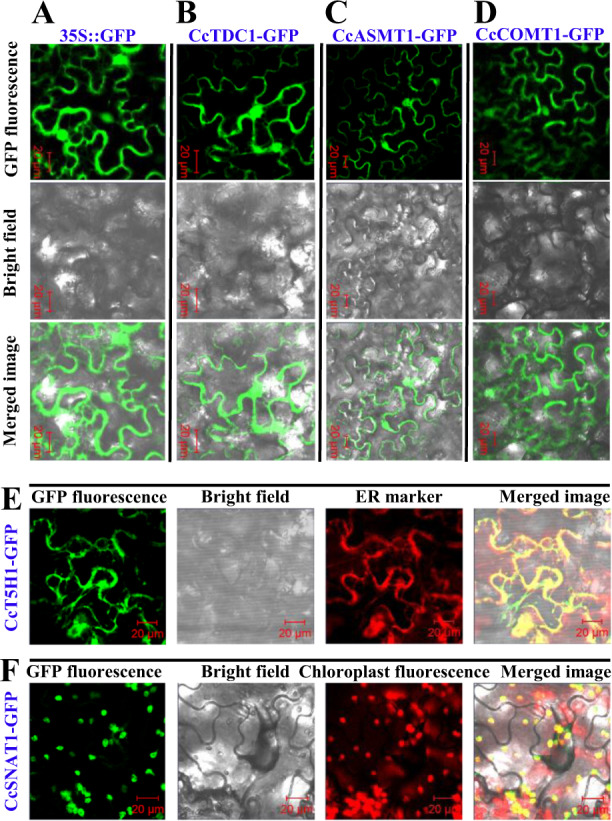
Subcellular localization of CcCOMT1, CcT5H1, CcSNAT1, CcTDC1, and CcASMT. **A** The GFP control vector was transiently expressed in tobacco leaves. **B** CcTDC1-GFP fusion plasmid was transiently expressed in tobacco leaves. **C** CcASMT1-GFP fusion plasmid was transiently expressed in tobacco leaves. **D** CcCOMT1-GFP fusion plasmid was transiently expressed in tobacco leaves. **E** CcT5H1-GFP fusion plasmid and ER marker were transiently coexpressed in tobacco leaves. **F** CcSNAT1-GFP fusion plasmid was transiently expressed in tobacco leaves. Proteins were localized with confocal fluorescence microscopy. Scale bars: 20 µm

To investigate the subcellular localization of the transcripts encoding enzymes, we constructed CcTDC1-GFP, CcT5H1-GFP, CcASMT1-GFP, CcCOMT1-GFP, and CcSNAT1-GFP fusion plasmids and transiently expressed them in tobacco leaves. Examination of epidermal cells by confocal microscopy showed that strong GFP signals of CcTDC1-GFP, CcCOMT1-GFP, and CcASMT1-GFP were present in the cytoplasm and nucleus, similar to those seen in the empty vector-GFP control (Fig. 4A-D). Therefore, our results suggest that CcTDC1, CcCOMT1, and CcASMT1 are soluble enzymes with no specific subcellular localization. As shown in Fig. 4E, the CcT5H1-GFP signal essentially overlapped with the OsPLA2α-RFP fusion protein, an ER marker. CcSNAT1-GFP colocalized with chloroplast autofluorescence (Fig. 4F). Thus, we concluded that CcT5H1 and CcSNAT1 were located in the ER and chloroplast, respectively.

### Screening of candidate transcriptional modulators involved in the melatonin biosynthesis pathway

**Fig. 5:**
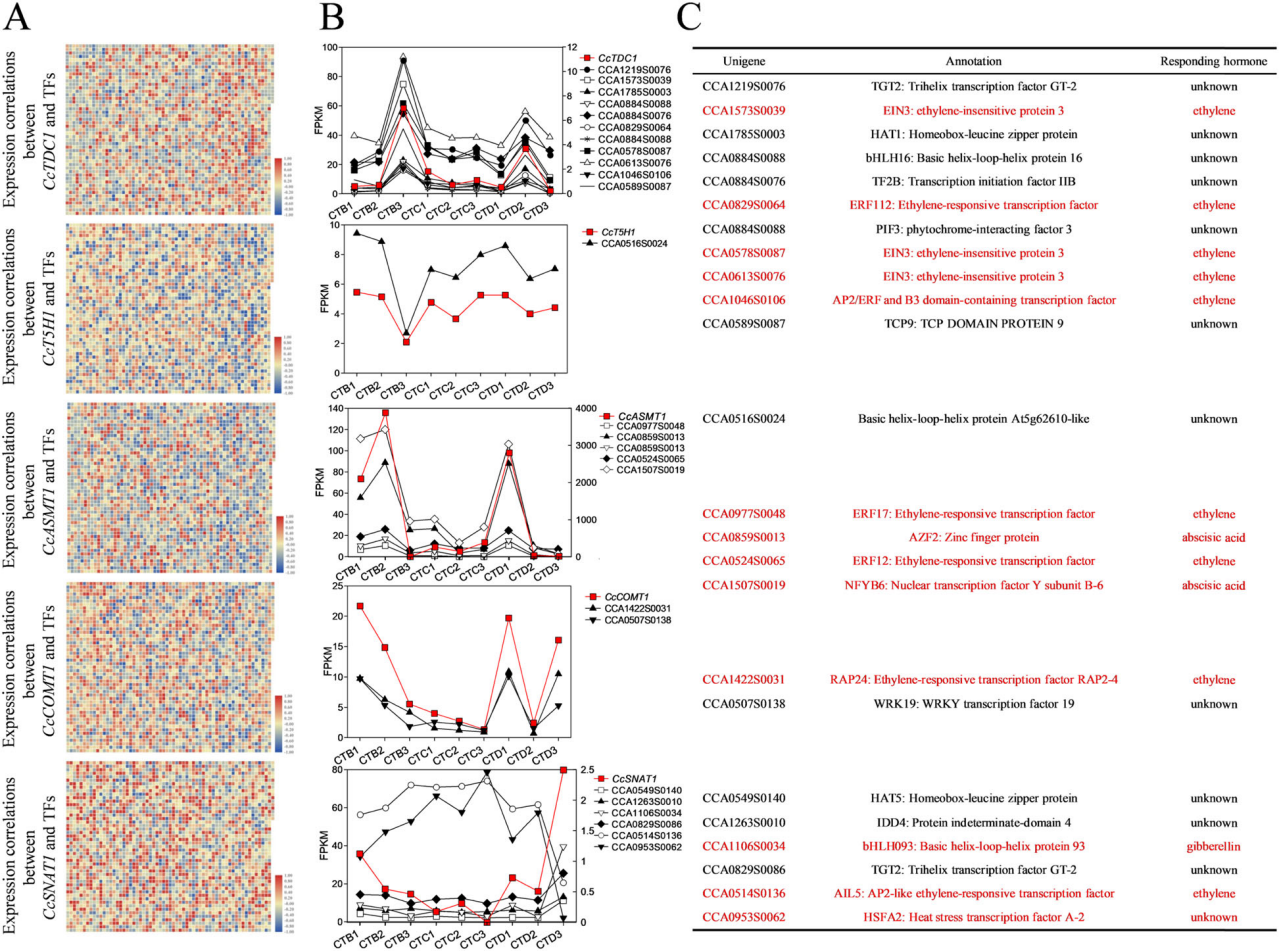
Screening of transcription factors coexpressed with genes encoding enzymes involved in the biosynthesis of melatonin from the transcriptome. **A** Pearson’s correlation analysis between the expression levels of genes encoding enzymes involved in the biosynthesis of melatonin and all transcription factors from the transcriptome. **B** Transcription factors significantly correlated with *CcTDC1*, *CcCOMT1*, *CcT5H1*, *CcSNAT1*, or *CcASMT1* with a Pearson’s correlation coefficient ≥ 0.95 and FPKM ≥ 10 were selected. The right *Y*-axis with a red line represents the *Y*-axis of *CcTDC1*, *CcASMT1*, or *CcSNAT1*. The transcription factors with green lines indicate that they have a negative correlation with *CcSNAT1*. **C** Annotations of transcription factors from **B**. Genes marked with a red color indicate that they are hormone-responsive transcription factors

Transcription factors are critical to regulate gene expression. To search for the transcription factors that were coexpressed with *CcTDC1*, *CcCOMT1*, *CcT5H1*, *CcSNAT1*, or *CcASMT1*, Pearson’s correlation analysis between the expression level of genes annotated as transcription factors and candidate genes was performed. The transcription factors that were significantly correlated with *CcTDC1, CcCOMT1*, *CcT5H1, CcSNAT1*, or *CcASMT1*, with Pearson’s correlation coefficients ≥ 0.95 and FPKM ≥ 10, were selected (Fig. 5A and B). Notably, we found that most of the selected transcription factors were phytohormone-responsive transcription factors (Fig. 5C). Five of the 11 transcription factors highly coexpressed with *CcTDC1* were ethylene-responsive genes. One of the two transcription factors highly coexpressed with *CcCOMT1* was an ethylene-responsive gene. Two of six transcription factors highly coexpressed with *CcSNAT1* were gibberellin-responsive genes, and one was an ethylene-responsive gene. Notably, all the selected transcription factors highly coexpressed with *CcASMT1* were phytohormone-responsive transcription factors. Of these transcription factors, two and two were ethylene- and ABA-responsive genes, respectively.

### Identification of candidate transcriptional modulators involved in the melatonin biosynthesis pathway

**Fig. 6:**
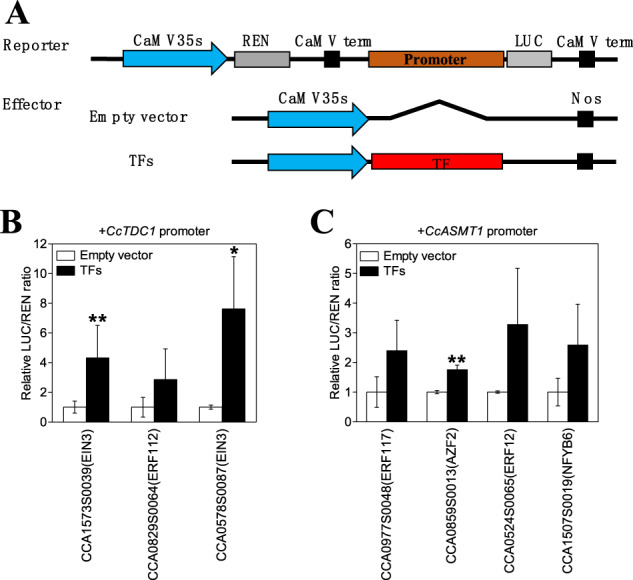
Identification of hormone-responsive transcription factors regulating melatonin synthase genes by dual-luciferase assay. **A** Diagrams of the reporter and effector vectors used in dual-luciferase assays. **B** CcEIN3-1 and CcEIN3-2 activate the promoter of *CcTDC1*. **C** CcAZF2 activates the promoter of *CcASMT1*. The LUC/REN value of the empty vector on each promoter was set as 1 as a calibrator. Each value represents the mean ± SD of three independent experiments, with three replicates in each experiment. Statistical significance was determined by Student’s two-tailed *t*-test (*P < 0.05, **P < 0.01)

A dual-luciferase assay was carried out to further identify which hormone-responsive transcription factors regulate melatonin synthase genes. The results showed that two transcription factors, CCA1573S0039 and CCA0578S0087, were both annotated as EIN3 (ethylene insensitive protein 3) and could activate the expression of CcTDC1 (Fig. 6B). For the four transcription factors that were coexpressed with *CcASMT1*, only one transcription factor, CCA0859S0013, annotated as AZF2 (ABA-responsive protein), could activate the expression of *CcASMT1* (Fig. 6C).

**Fig. 7:**
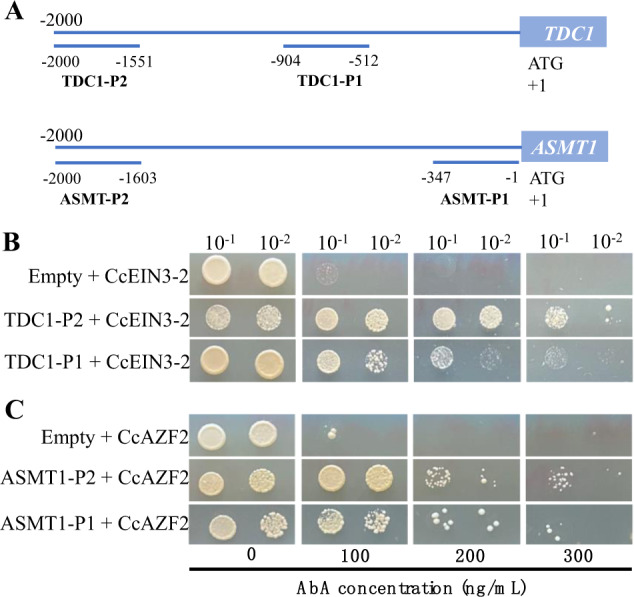
CcEIN3 and CcAZF2 activate *CcTDC1* and *CcASMT1* by directly binding to their promoters, respectively. **A** Schematic diagram of the bait fragments used to construct the reporter vectors in the yeast one‐hybrid assay. **B** Yeast one‐hybrid assay for CcEIN3-2 and promotor fragments of *CcTDC1*. **C** Yeast one‐hybrid assay for CcAZF2 and promoter fragments of *CcASMT1*. Each pair of plasmids was cointroduced into yeast strain Y1H Gold and cultured on SD medium without Leu containing different concentrations of AbA at 30 °C for 72 h

To further verify whether CcEIN3 and CcAZF2 regulate *CcTDC1* and *CcASMT1* by directly binding to their promoters, the yeast one-hybrid method was used. EIN3 and AZF2 can recognize EIN3-binding sites (ATGTAT, ATACAT, CTACAT, or ATGTAC)^45^ and A(G/C)T-box^46^, respectively. We found that there are many sites similar to EIN3-binding sites and many A(G/C)T repeat sequences in the 2000 bp length of *CcTDC1* and *CcASMT1* promoter sequences upstream of the *CcTDC1* and *CcASMT1* translation start codons, respectively (Supplementary Fig. S3). We selected promoter fragments with more potential *cis*-acting elements distributed on them for further yeast one-hybrid analysis (Fig. 7A). The results of yeast one-hybrid assays showed that CcEIN3 and CcAZF2 could bind to the *CcTDC1* and *CcASMT1* promoters to activate *AUR1-C* expression in yeast (Fig. 7B and C).

To verify whether CCA0859S0013 (named *CcAZF2*) and its homologs respond to ABA and whether CCA1573S0039 (named *CcEIN3-1*), CCA0578S0087 (named *CcEIN3-2*) and their homologs respond to ethylene in hickory embryos, different concentrations of ABA (50, 100, or 150 mg/L) and Ethrel (500, 1000, or 1500 mg/L) were sprayed onto hickory fruits for 7 days from July 24 to July 30. Water was used as a control. The real-time RT-PCR results showed that 100 mg/L and 150 mg/L ABA increased the expression level of *CcAZF2* (Supplementary Fig. S4A). The expression of both *CcEIN3-1* and *CcEIN3-2* was upregulated by Ethrel at concentrations of 500 and 1500 mg/L (Supplementary Fig. S5A and B). The expression of the homologs of *CcAZF2*, the homologs of *CcEIN3-1*, and the candidate pathway genes was also significantly regulated by ABA and Ethrel (Supplementary Fig. S6). Phylogenetic analysis results showed that CcAZF2 was most closely clustered with XP_018844631.1 (*Juglans regia*) (Supplementary Fig. S4B), and CcEIN3-1 and CcEIN3-2 were most closely clustered with XP_018850150.1 (*Juglans regia*) and XP_018846243.1 (*Juglans regia*) (Supplementary Fig. S5C and D), respectively. The protein domains of each transcription factor were predicted by PROSITE. CcAZF2 protein sequences contained two highly conserved zinc finger C_2_H_2-_type domains, which were similar to AtAZF2 (Supplementary Fig. S4C). Each of the CcEIN3 protein sequences contained a heme-binding NEAT domain that was similar to AtEIN3 (Supplementary Fig. S5E).

## Discussion

Melatonin, a multifunctional hormone, is involved in improving the tolerance of biotic and abiotic stresses in plants and regulating plant development and growth^47^. In this study, we found that hickory nuts contain upto 485–839 pg/g melatonin. However, the molecular regulatory mechanism underlying the high level of melatonin synthesis is still unclear. The identification of the biosynthetic pathways of bioactive components and characterization of the related genes are essential for understanding the molecular regulatory mechanism.

In this study, the melatonin biosynthesis pathway in hickory was proposed for the first time by combined metabolome and transcriptome analysis. Transcriptome sequencing-generated clean reads were mapped to the hickory reference genome. From the annotated genes, 9, 11, 1, 7, and 1 were annotated as TDC, T5H, SNAT, COMT, and ASMT, respectively. However, no gene was annotated as TPH. Similarly, genes encoding the enzymes of TDC, T5H, SNAT, COMT, and ASMT in various plant species have also been identified, except the putative gene encoding TPH^20^. TPH is the first enzyme in the melatonin biosynthesis pathway in animals and uses tryptophan as a substrate to synthesize 5-hydroxytryptophan^20,48^. The cloning of TPH coding genes and determination of corresponding TPH enzyme activities in plants have not been reported.

Overall, 608 metabolites were identified by UPLC-ESI-MS/MS. To investigate the metabolic components involved in the melatonin biosynthesis pathway, we focused on tryptophan and the class of tryptamine derivatives. All metabolites mentioned in Fig. 2 were detected, except 5-methoxytryptamine. As tryptophan and 5-hydroxytryptophan, which are the substrate and product of TPH, respectively, were detected, there must be a gene in hickory that encodes an enzyme that performs the same function as TPH in animals. Some evidence suggests that TPH-like genes exist in plants, although no animal TPH homologs have been found in plant genomes. For example, the seeds of *Griffonia simplicifolia* are rich in 5-hydroxytryptophan^49,50^, and the soluble fraction of extracts from rice roots showed tetrahydropterin-dependent amino-acid hydroxylase activity, which is similar to TPH^51^. Although the suppression of *T5H* can reduce serotonin levels, it has been observed that suppression lines accumulate more melatonin than control lines, suggesting that a putative TPH is a key target for promoting melatonin synthesis in plants^52^.

It was reported that COMT/ASMT can catalyze *N*-acetylserotonin to synthesize melatonin and/or methylate serotonin into 5-methoxytryptamine in the cytoplasm^19^. In this study, 5-methoxytryptamine was not detected, suggesting that COMT/ASMT may have no catalytic activity against serotonin, resulting in the inability to methylate serotonin into 5-methoxytryptamine, or the content of 5-methoxytryptamine was too low to be detected. In *Arabidopsis*, AtSNAT had a 23-fold higher catalytic efficiency toward 5-methoxytryptamine than toward serotonin^53^. CcSNAT in hickory is probably similar to AtSNAT in Arabidopsis, which has higher catalytic efficiency toward 5-methoxytryptamine. Therefore, the content of 5-methoxytryptamine in embryos of hickory is likely too low to be detected.

The localization of a protein is very important for its function. In this study, the candidate genes encoding TDC, T5H, SNAT, COMT, and ASMT in hickory were first identified by phylogenetic analysis and amino-acid sequence alignment. However, it is not clear whether these candidate genes encoding proteins, such as their plant counterparts, are located in a specific subcellular component to perform their functions. Previous studies have demonstrated the subcellular localization of enzymes involved in melatonin synthesis. Both AtCOMT and OsCOMT lack leader or transit sequences and are localized in the cytoplasm^54,55^. The fluorescence of Arabidopsis SNAT and rice SNAT1 and SNAT2 merged with the fluorescence of chlorophyll, indicating that they are localized to chloroplasts^53,56,57^. In rice, the fluorescence of OsASMT1-mCherry, OsASMT2-mCherry, and OsASMT3-mCherry was colocalized with the fluorescence of cytoplasmic GFP^58^. In *Catharanthus roseus* and *Tabernaemontana divaricata*, TDC is localized in the cytoplasm^59,60^. The localization of MeTDC2, MeASMT2, and MeASMT3 from cassava was investigated using transient expression in tobacco leaves, and the results showed that they were localized in both the cytoplasm and nucleus^61,62^. T5H has been shown to be localized in the endoplasmic reticulum^63^. Similarly, our results showed that CcTDC1, CcCOMT1, and CcASMT1 were localized in the cytoplasm and nucleus, and CcT5H1 and CcSNAT1 were localized in the ER and chloroplast, respectively (Fig. 4). These results further suggested that CcTDC1, CcT5H1, CcSNAT1, CcCOMT1, and CcASMT1 in hickory, similar to their homologous proteins in other plants, function as melatonin biosynthesis pathway enzymes.

**Fig. 8:**
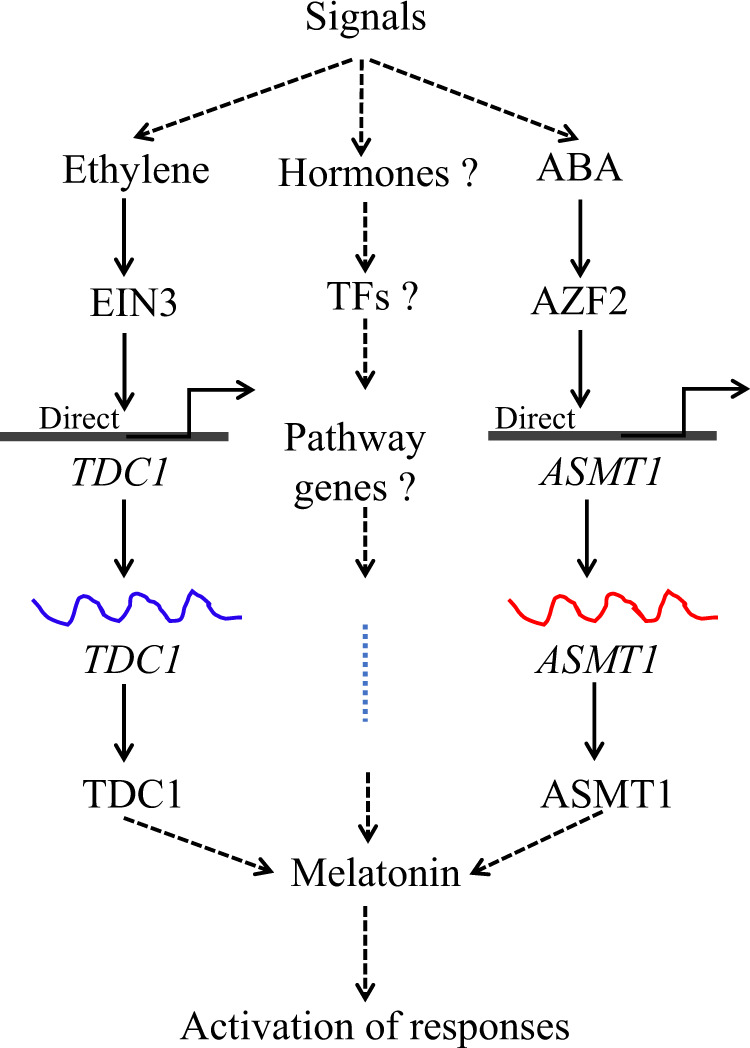
A new hypothetical model of melatonin biosynthesis regulated by other phytohormones. Some upstream signals cause hickory to accumulate more ethylene and abscisic acid, which in turn upregulate the expression of CcEIN3 and CcAZF2, respectively. EIN3 and AZF2 directly promote the expression of *CcTDC1* and *CcASMT1*, respectively, and increase the melatonin content, thus inducing the activation of signal responses

Transcription factors could bind to the *cis*-acting element on the promoter of their target genes to regulate gene expression. In plants, transcription factors play vital roles in various biological processes, including developmental regulation, defense induction, and stress responses^26,64–67^. However, only a few transcription factors were found to be involved in the regulation of melatonin synthesis. By using chromatin immunoprecipitation, an electrophoretic mobility shift assay, and activation of promoter activity, Wei et al. found that MeWRKY79 and MeHsf20 could target the W-box and heat-stress elements (HSEs), respectively, in the promoter of *MeASMT2* in cassava. MeWRKY79- and MeHsf20-silenced plants showed lower MeASMT2 transcripts and less melatonin accumulation, leading to disease sensitivity^37^. MeWRKY20/75 can interact with MeTDC2/MeASMT2/3 to form a protein complex to further regulate melatonin levels^39^. Both an in vitro electrophoretic mobility shift assay and in vivo chromatin immunoprecipitation coupled with qPCR analysis revealed that the transcription factor HsfA1a binds to the *COMT1* gene promoter and acts as a positive regulator of *COMT1* to promote melatonin accumulation and increase Cd tolerance^38^. MeRAV1 and MeRAV2 positively regulate *MeTDC2*, *MeT5H*, and *MeASMT1* by directly binding to their promotors in cassava^35^. However, the transcription factors that regulate melatonin synthesis in hickory have not been identified. Therefore, finding transcription factors related to melatonin biosynthesis in hickory is essential for investigating its melatonin biosynthesis regulatory mechanisms. In this study, we found that most of the transcription factors highly coexpressed with melatonin synthase genes were ethylene-, ABA-, or GA-responsive transcription factors, suggesting that these phytohormone-responsive transcription factors may regulate melatonin biosynthesis by inducing the expression of melatonin biosynthesis genes. The characterization of melatonin as a regulatory factor involved in the expression of enzymes and regulatory element of plant hormones is an interesting and controversial research direction. Studies have shown that exogenous melatonin regulates genes associated with plant hormones, from genes involved in IAA, CKs, Gas, ABA, JA, and ethylene biosynthesis or catabolism to genes encoding auxin carriers, such as PINs, ethylene-related proteins (e.g., NR and ETR4), ABA receptors (PYL8), and signal transduction elements^23^. However, the effects of other plant hormones on melatonin have not been reported. Our results proposed a new hypothetical model of melatonin biosynthesis regulated by other phytohormones. As shown in Fig. 8, some upstream signals result in increased ethylene and ABA accumulation in hickory, which could further upregulate the expression of CcEIN3 and CcAZF2, respectively. EIN3 and AZF2 directly bind to the promoters of *CcTDC1* and *CcASMT1* and promote the expression of *CcTDC1* and *CcASMT1*, respectively. Finally, the melatonin content is increased, and subsequent activation of the signal response is induced, which needs to be verified by further experiments. It is also possible that other hormones or transcription factors, which we have not yet found, are involved in melatonin biosynthesis.

In summary, we generated comprehensive metabolome and transcriptome databases from hickory using UPLC-ESI-MS/MS and high-throughput RNA sequencing. We proposed a melatonin biosynthesis pathway in hickory through combined metabolome and transcriptome analysis. Most notably, the metabolic components (L-tryptophan, L-tryptamine, 5-hydroxytryptophan, serotonin, *N*-acetylserotonin, and melatonin) involved in the melatonin biosynthesis pathway, and the candidate genes encoding TDC, T5H, COMT, SNAT, and ASMT were identified. Further identification of the gene encoding TPH and confirmation of whether 5-methoxytryptamine can be produced in hickory are needed to better understand the melatonin biosynthesis pathway. One ABA-responsive transcription factor (CcAZF2) and two ethylene-responsive transcription factors (CcEIN3-1 and CcEIN3-2) were shown for the first time to activate the transcription of CcASMT1 and CcTDC1, respectively. Our results revealed novel melatonin biosynthesis regulatory mechanisms and enriched our understanding of the interaction between melatonin and other plant hormones.

## Supplementary information

Table S1 Primers used in this study

Table S2 All the annotated metabolites in embryos of hickory

Table S3 Summary of transcriptome sequencing output data for hickory

Fig. S1 Validation of RNA-Seq data by qRT-PCR

Fig. S2 Phylogenetic analysis of proteins potentially encoding melatonin biosynthesis enzymes from hickory and known proteins controlling melatonin biosynthesis from a range of plants

Fig. S3 The promoter sequences of CcTDC1 (A) and CcASMT1 (B)

Fig. S4 qRT-PCR analysis, phylogenetic analysis and alignment of the amino-acid sequence of CcAZF2

Fig. S5 qRT-PCR analysis, phylogenetic analysis and alignment of the amino-acid sequence of CcEIN3-1, CcEIN3-2

Fig. S6 qRT-PCR analysis of expression patterns of hormone response genes and melatonin synthesis genes under different hormone treatment with different concentrations and time
