# Supplementary material for: Metabolite profiling and transcriptome analyses reveal novel regulatory mechanisms of melatonin biosynthesis in hickory
Source: Hortic Res. 2021 Sep 1;8:196. doi: 10.1038/s41438-021-00631-x (PMC8408178; doi:10.1038/s41438-021-00631-x)
Supplement: Supplementary file 1 — Table S1 Primers used in this study [file 41438_2021_631_MOESM1_ESM.docx]

**Table S1** Primers used in this study.

| Application | Primer name | Primer sequence 5‘→3’ |
| --- | --- | --- |
| Subcelluer localization | CcCOMT1-InFu-GFP-BamHI-F | GAGCTCGGTACCCGGGGATCCATGGGTTCAACCGGCG |
|  | CcCOMT1-InFu-GFP-SalI-R | TCGCCCTTGCTCACCATGTCGACAAGCTTTTTAATGAATTCCATAATATATG |
|  | CcTDC1-InFu-GFP-BamHI-F | GAGCTCGGTACCCGGGGATCCATGGGTAGCCTTGATTGCTC |
|  | CcTDC1-InFu-GFP-SalI-R | TCGCCCTTGCTCACCATGTCGAC AACACCATTTAGAACAGCATATGCC |
|  | CcT5H1-InFu-GFP-BamHI-F | GAGCTCGGTACCCGGGGATCCATGGAGTCGATTACTTCGCAA |
|  | CcT5H1-InFu-GFP-SalI-R | TCGCCCTTGCTCACCATGTCGACACCCTTGAACTCATACTGCGG |
|  | CcASMT1-InFu-GFP-BamHI-F | GACGAGCTGTACAAGGGATCCATGGGAAGTACACAGAGATC |
|  | CcASMT1-InFu-GFP-SalI-R | TCGCCCTTGCTCACCATGTCGACTTACTCTTTCCCCGTTGTAG |
|  | CcSNAT1-InFu-GFP-BamHI-F | GAGCTCGGTACCCGGGGATCCATGCTTGTCCGTGGCATCATC |
|  | CcSNAT1-InFu-GFP-SalI-R | TCGCCCTTGCTCACCATGTCGACTTTCTTGTTCTTTTTGGGCTTTGTG |
|  |  |  |
| qRT-PCR | CcASMT-qPCR-1-F | GACCTTCCACACGTCGTAGCTA |
|  | CcASMT-qPCR-1-R | CGCTCCAACCGTGTAAAACCAA |
|  | CcASMT-qPCR-2-F | AAAGTGGGTTCTGCACGATTGG |
|  | CcASMT-qPCR-2-R | TATCACCTTCCCTTTGCCCTCC |
|  | CcCOMT-qPCR-1-F | CCTTCCAGTAGCACCAGACACT |
|  | CcCOMT-qPCR-1-R | TGAAATCCAGCCCCTTTAGCCA |
|  | CcCOMT-qPCR-2-F  CcCOMT-qPCR-2-R  CcCOMT-qPCR-3-F  CcCOMT-qPCR-3-R | CGACATGGACGGATGGTGAAGT  GGCTCACAGACGATCAATTTCCC  TACCCAGGCACAGACGAAAG  CGCCACCAACATCAACCAAC |
|  | CcCOMT-qPCR-4-F | CGCAAGGTAAGGAGAGGACAGA |
|  | CcCOMT-qPCR-4-R | CAAAGCCTGCCACCTGAATACC |
|  | CcCOMT-qPCR-5-F  CcCOMT-qPCR-5-R | CACGTTGCAGGAGACATGTT  TATCACTTTCCCACGCTCCG |
|  | CcSNAT-qPCR-1-F | GCTGTCATGGAGAGGTTGTTGG |
|  | CcSNAT-qPCR-1-R | GAGTACACCATTCCCCGGATCC |
|  | CcSNAT-qPCR-2-F | GCATGCGGATTTGGAGATGACA |
|  | CcSNAT-qPCR-2-R  CcT5H-qPCR-1-F  CcT5H-qPCR-1-R | ATTGCCTGGGTGAGAAGTGGAA  AGGTGAGAAGAGTGGTGGGA  CGAAAGAGGAGCTGGTGGAT |
|  | CcT5H-qPCR-2-F | TGCCAAGGATCAGGACTTCAGG |
|  | CcT5H-qPCR-2-R | CCAATGCAATCTCTACCGTGGC |
|  | CcTDC-qPCR-1-F | GTGTGCTTCCGTTTGAACCCAT |
|  | CcTDC-qPCR-1-R  CcTDC-qPCR-2-F  CcTDC-qPCR-2-R | TCACCCAATCGAGTAGCTTCCG  GGCAGGCACCACTCATCTAT  CCATTCGGGTCTCAAGCTCA |
|  | CcActin-qPCR-F | GCTGAACGGGAAATTGTC |
|  | CcActin-qPCR-R  CcEIN3-3-DL-F  CcEIN3-3-DL-R  CcEIN3-4-DL-F  CcEIN3-4-DL-R  CcEIN3-6-DL-F  CcEIN3-6-DL-R  CcEIN3-7-DL-F  CcEIN3-7-DL-R  CcEIN2-1-DL-F  CcEIN2-1-DL-R  CcEIN2-2-DL-F  CcEIN2-2-DL-R  CcEIN4-1-DL-F  CcEIN4-1-DL-R  CcAZF3-DL-F  CcAZF3-DL-R  ASMT-2-DL-ABA-F  ASMT-2-DL-ABA-R  TDC-2-DL-ETH-F  TDC-2-DL-ETH-R  TDC-10-DL-ETH-F  TDC-10-DL-ETH-R | AGAGATGGCTGGAAGAGG  GCGATGAGTGAGGCAGATGA  GGGGTCACAGTGTTGCATCA  CATGTATGTGGACGGGAGGC  ACAATCTGAGACTGCTGCCA  GGCGTCCAGTGATTTGAACG  CGGACTTCGCTGTAAGGACA  TGAGGCCCCTGGAGAAAGAT  TCCCGTCCACATACATGCTC  GGATCAAGGGCAGGACATCC  TGTCAGCTACGCCAAACTGT  GGATGTGGTTCTGGGAGTGG  AACTCCTCCAGACAGCAAGC  GCTAGTGCCGAGGAACATGT  GCTCATCTGCCATTCCTTGC  GCAACGCTAGAAACTGCTCC  CAGCGCCGTTCCTGTAAAAG  ATACCAATGCCAACGCCAACAG  AAAGCTGGCTCCTCTTCCTCTC  TGGCCCGAAAGTTGTATCCTGA  AGCCTATCTCTTGGTCCAGCAG  CCTGATAGATGCCCACCTGTGT  AGCAGCCCCGATATTGAAGAGG |
| Double luciferase reporter experiment  Y1H experiment | ASMT-TF-1-57-F | GAGCTCGGTACCCGGGGATCCATGGTGAAGCAGACCACTG |
|  | ASMT-TF-1-59-R | TCGCCCTTGCTCACCATGTCGACCTAGAAATTCCAAAGGAATGACG |
|  | ASMT-TF-2-60-F | GAGCTCGGTACCCGGGGATCCATGGCCTTAGAAGCTCTGAATTC |
|  | ASMT-TF-2-59-R  ASMT-TF-3-60-F  ASMT-TF-3-60-R  ASMT-TF-4-57-F  ASMT-TF-4-57-R  ASMT-TF-5-60-F  ASMT-TF-5-59-R  TDC-TF-1-61-F  TDC-TF-1-60-R  TDC-TF-2-59-F  TDC-TF-2-61-R  TDC-TF-3-57-F  TDC-TF-3-58-R  TDC-TF-4-61-F  TDC-TF-4-60-R  TDC-TF-5-58-F  TDC-TF-5-58-R  TDC-TF-6-60-F  TDC-TF-6-61-R  TDC-TF-7-59-F  TDC-TF-7-60-R  TDC-TF-8-58-F  TDC-TF-8-60-R  TDC-TF-10-60-F  TDC-TF-10-60-R  CcTDC-PRO-pGREEN-SalI-F  CcTDC-PRO-pGREEN-HinⅢ-R  CcASMT-PRO-pGREEN-SalI-F  CcASMT-PRO-pGREEN-HiⅢ-R  TDC-1-410-F-SacI  TDC-1-410-R-Xho  TDC-365-784-SacI  TDC-365-784-Xho  TDC-748-1150-SacI  TDC-748-1150-Xho  TDC-1097-1489-SacI  TDC-1097-1489-Xho  TDC-1375-1754-SacI  TDC-1375-1754-Xho  TDC-1729-2000-SacI  TDC-1729-2000-Xho  ASMT-1-398-F-SacI  ASMT-1-398-R-Xho  ASMT-350-738-F-SacI  ASMT-350-738-R-Xho  ASMT-679-1076-F-SacI  ASMT-679-1076-R-Xho  ASMT-991-1359-F-SacI  ASMT-991-1359-R-Xho  ASMT-1269-1689-F-SacI  ASMT-1269-1689-R-Xho  ASMT-1654-2000-F-SacI  ASMT-1654-2000-R-Xho  CcAZF1-pGADT7-EcoRI-F  CcAZF1-pGADT7-Sac-R  CcEIN3-1-pGADT7-EcoRI-F  CcEIN3-1-pGADT7-Sac-R  CcEIN3-2-pGADT7-EcoRI-F  CcEIN3-2-pGADT7-Sac-R | TCGCCCTTGCTCACCATGTCGACTCAATCTTGGGCAGAAACC  GAGCTCGGTACCCGGGGATCCATGGCCTTAGAAGCTCTGAATTC  TCGCCCTTGCTCACCATGTCGACTCACGCCGCTGTTGG  GAGCTCGGTACCCGGGGATCCATGGTGAAGACAGAGCAGAAG  TCGCCCTTGCTCACCATGTCGACTCAGCGGAAGCTCCATAG  GAGCTCGGTACCCGGGGATCCATGCCGATTGCCAACG  TCGCCCTTGCTCACCATGTCGACTCATTTATACTGGGCATGTGC  GAGCTCGGTACCCGGGGATCCATGAGGAAGAAGAAGAAGAAGAAATTG  TCGCCCTTGCTCACCATGTCGACTCAACCCACAGTTGCCATT  GAGCTCGGTACCCGGGGATCCATGGGAACCTTTGAAGACATG  TCGCCCTTGCTCACCATGTCGACTCAAAGGTACCACAGGGAGATG  GAGCTCGGTACCCGGGGATCCATGGTTGAGAAGGAAGATCTTG  TCGCCCTTGCTCACCATGTCGACTCAGATCAATATTTTTTTTTTCTTTTTATAG  GAGCTCGGTACCCGGGGATCCATGGTTGAGAAGGAAGATCTTGG  TCGCCCTTGCTCACCATGTCGACTTATTTTTTACTAAGTTCCTTGAAAAGAAAG  GAGCTCGGTACCCGGGGATCCATGAAGCCGAGTTTGAAGG  TCGCCCTTGCTCACCATGTCGACTTAGCTCTTAGAACTAGAAGGTGGAG  GAGCTCGGTACCCGGGGATCCATGAAAAGAATCCTGAAATATGAAGC  TCGCCCTTGCTCACCATGTCGACTCAAGGGCTGCAAAGGTTC  GAGCTCGGTACCCGGGGATCCATGACGACAGATATGGTGTCG  TCGCCCTTGCTCACCATGTCGACTTAACTGGAAGAGCTAGGATAGTGAAC  GAGCTCGGTACCCGGGGATCCATGTGCTTGCTTATAAAGGTGG  TCGCCCTTGCTCACCATGTCGACTTAACTGGAAGAGCTAGGATAGTGAAC  GAGCTCGGTACCCGGGGATCCATGGGAATCTTTGAAGATATGGG  TCGCCCTTGCTCACCATGTCGACTCAAAGGTACCACGAGGAGATATC  GGGCCCCCCCTCGAGGTCGACAAATCACTTCAACGATCATAAATTGGG  CAGGAATTCGATATCAAGCTTATCAGGGTTTGATTGGATCGACTATC  GGGCCCCCCCTCGAGGTCGACTGCAACTACATCAGCTAATAG  CAGGAATTCGATATCAAGCTTCTCTTTCTCTCTCTGTTTGAGC  GAAAAGCTTGAATTCGAGCTCAAATCACTTCAACGATCATAAATTGG  ATACAGAGCACATGCCTCGAGCACTTGCTTAACTATTAATTAAAAAATTAAAATATTC  GAAAAGCTTGAATTCGAGCTCGTACCGTTTGAATATTTTAATTTTTTAATTAATAG  ATACAGAGCACATGCCTCGAGGCCGACTGAATTATTAGAATTAATATTG  GAAAAGCTTGAATTCGAGCTCGCGGGTAACAATATTAATTCTAATAATTC  ATACAGAGCACATGCCTCGAGCCAGACAAGAACGACATATGTTAAATC  GAAAAGCTTGAATTCGAGCTCGGGCAGGACCGATCCT  ATACAGAGCACATGCCTCGAGGGATTGGGGGTTCTTGAGAT  GAAAAGCTTGAATTCGAGCTCCGTATTGATAGATGTCAGGTATTTTTTTAAG  ATACAGAGCACATGCCTCGAGGCATTGTCCGGTCAAGTTAAC  GAAAAGCTTGAATTCGAGCTCCGCCGGTTAACTTGACCG  ATACAGAGCACATGCCTCGAGATCAGGGTTTGATTGGATCG  GAAAAGCTTGAATTCGAGCTCTGCAACTACATCAGCTAATAGTTTAATC  ATACAGAGCACATGCCTCGAGGTTGTAATGATGATATGAGATGAAAAATTTTC  GAAAAGCTTGAATTCGAGCTCGACCCCGTTTGGATAGTGAA  ATACAGAGCACATGCCTCGAGGGGCATGATCTTCCAAGC  GAAAAGCTTGAATTCGAGCTCGTGGCAATCATTCTTGAAAAATG  ATACAGAGCACATGCCTCGAGCAGAGACATGTCTAGATATTTTAAAATTTT  GAAAAGCTTGAATTCGAGCTCGAACATGGCCCCCTCAA  ATACAGAGCACATGCCTCGAGCTCTTATGTAAATAAAAAAGAGATTGAAAAATC  GAAAAGCTTGAATTCGAGCTCGATAGATTATTTAAGTTCGATAATCTATAAGAAAAATAG  ATACAGAGCACATGCCTCGAGGGCAGCGGCGGAAC  GAAAAGCTTGAATTCGAGCTCGTCCCCTTCGATACAATTGC  ATACAGAGCACATGCCTCGAGCTCTTTCTCTCTCTGTTTGAGCAAG  GCCATGGAGGCCAGTGAATTCATGGCCTTAGAAGCTCTGAATTC  ATTCATCTGCAGCTCGAGCTCTCAATCTTGGGCAGAAACC  GCCATGGAGGCCAGTGAATTCATGGGAACCTTTGAAGACATG  ATTCATCTGCAGCTCGAGCTCTCAAAGGTACCACAGGGAGATG  GCCATGGAGGCCAGTGAATTCATGGGAATCTTTGAAGATATGGG  ATTCATCTGCAGCTCGAGCTCTCAAAGGTACCACGAGGAGATATC |
