# Supplementary material for: Metabolite profiling and transcriptome analyses reveal novel regulatory mechanisms of melatonin biosynthesis in hickory
Source: Hortic Res. 2021 Sep 1;8:196. doi: 10.1038/s41438-021-00631-x (PMC8408178; doi:10.1038/s41438-021-00631-x)
Supplement: Supplementary file 3 — Table S3 Summary of transcriptome sequencing output data for hickory [file 41438_2021_631_MOESM3_ESM.docx]

**Table S3** Summary of transcriptome sequencing output data for hickory.

| Sample | Raw Reads | Clean Reads | Clean Base (G) | Error Rate (%) | Q20 (%) | Q30 (%) | GC Content (%) | Reads mapped (%) |
| --- | --- | --- | --- | --- | --- | --- | --- | --- |
| CTB1 | 66939670 | 64385870 | 9.66 | 0.03 | 96.89 | 91.84 | 48.04 | 96.28 |
| CTB2 | 47789286 | 45961654 | 6.89 | 0.03 | 97.57 | 93.29 | 48.18 | 96.83 |
| CTB3 | 57410716 | 55577538 | 8.34 | 0.03 | 97.42 | 93.02 | 47.17 | 96.70 |
| CTC1 | 55847534 | 53736050 | 8.06 | 0.03 | 97.27 | 92.65 | 46.71 | 95.83 |
| CTC2 | 58237554 | 56658974 | 8.50 | 0.03 | 97.56 | 93.29 | 46.50 | 96.65 |
| CTC3 | 61740324 | 59943724 | 8.99 | 0.03 | 97.46 | 93.06 | 47.25 | 96.48 |
| CTD1 | 56849708 | 54579448 | 8.19 | 0.03 | 97.32 | 92.74 | 47.85 | 96.60 |
| CTD2 | 53335050 | 51368800 | 7.71 | 0.03 | 97.56 | 93.26 | 46.64 | 96.94 |
| CTD3 | 52793736 | 50372684 | 7.56 | 0.03 | 97.24 | 92.57 | 46.13 | 96.41% |
