# Supplementary material for: Metabolite profiling and transcriptome analyses reveal novel regulatory mechanisms of melatonin biosynthesis in hickory
Source: Hortic Res. 2021 Sep 1;8:196. doi: 10.1038/s41438-021-00631-x (PMC8408178; doi:10.1038/s41438-021-00631-x)
Supplement: Supplementary file 4 — Fig. S1 Validation of RNA-Seq data by qRT-PCR [file 41438_2021_631_MOESM4_ESM.docx]

**Fig. S1** Validation of RNA-Seq data by qRT-PCR. (A) to (N) Correlation of gene expression levels between RNA-seq data and qRT-PCR analysis. The sequences included from (A) to (M) are as follows: CCA1453S0002, CCA0568S0058, CCA0646S0015, CCA0898S0029, CCA0743S0031, MSTRG.1826, MSTRG.22548, CCA1151S0049, CCA0903S0007, CCA0803S0009, CCA0918S0075 , CCA0957S0018, CCA0982S0017.
