# Supplementary material for: Metabolite profiling and transcriptome analyses reveal novel regulatory mechanisms of melatonin biosynthesis in hickory
Source: Hortic Res. 2021 Sep 1;8:196. doi: 10.1038/s41438-021-00631-x (PMC8408178; doi:10.1038/s41438-021-00631-x)
Supplement: Supplementary file 5 — Fig. S2 Phylogenetic analysis of proteins potentially encoding melatonin biosynthesis enzymes from hickory and known proteins controlling melatonin biosynthesis from a range of plants [file 41438_2021_631_MOESM5_ESM.docx]

**Fig. S2** Phylogenetic analysis of proteins potentially encoding melatonin biosynthesis enzymes from hickory and known proteins controlling melatonin biosynthesis from a range of plants. (A) Phylogenetic tree of *TDC* from *C. cathayensis* and other plants. The sequences included are as follows: *Catharanthus roseus* (CAA47898.1), *Rauvolfia verticillate* (ABP96805.1), *Ophiorrhiza prostrata* (ABU40982.1), *Camptotheca acuminate* (AAB39709.1), *Oryza sativa* (XP_015648768.1, XP_015644906.1 and NM001067504) and *Juglans regia* (XP_035550439.1). (B) Phylogenetic tree of *T5H* from *C. cathayensis* and other plants. The sequences included are as follows: *Juglans regia* (XP_035540507.1) and *Oryza sativa* (XP_015618264.1). (C) Phylogenetic tree of *ASMT* from *C. cathayensis* and other plants. The sequences included are as follows: *Juglans regia* (XP_018823832.2), Arabidopsis (NP_195242.1) and *Oryza sativa* (XP_015610997.1, Q8VWJ6.1, Q8VWG4.1),. (D) Phylogenetic tree of *COMT* from *C. cathayensis* and other plants. The sequences included are as follows: *Juglans regia* (XP_018828596.1), *Saccharum officinarum* (CAA13175.1), *Panicum virgatum* (ADX98508.1), *Sorghum bicolor* (AAO43609.1), Maize (Q06509.1), *Lolium perenne* (AAD10253.1), *Medicago sativa* (AAB46623.1) and Arabidopsis (AK064768). (E) Phylogenetic tree of *SNAT* from *C. cathayensis* and other plants. The sequences included are as follows: *Oryza sativa* (XP_015637887.1, XP_015648698.1), Arabidopsis (NP_564387.1), *Spirulina subsalsa* (WP_017304728.1), *Populus trichocarpa* (XP_002312367.2), *Vitis vinifera* (XP_002271276.1), *Brachypodium distachyon* (XP_003571288.1) and *Juglans regia* (XP_018818545.1).
