# Supplementary material for: Metabolite profiling and transcriptome analyses reveal novel regulatory mechanisms of melatonin biosynthesis in hickory
Source: Hortic Res. 2021 Sep 1;8:196. doi: 10.1038/s41438-021-00631-x (PMC8408178; doi:10.1038/s41438-021-00631-x)
Supplement: Supplementary file 6 — Fig. S3 The promoter sequences of CcTDC1 (A) and CcASMT1 (B) [file 41438_2021_631_MOESM6_ESM.docx]

**Fig. S3** The promoter sequences of *CcTDC1* (A) and *CcASMT1* (B). The position of nucleotide in the promoter is opposite to the translational start site (ATG) that was marked as +1. The sites similar to EIN3-binding sites (EBS-like) in *CcTDC1* promotor and A(G/C)T repeats in *CcASMT1* promotor were boxed.
