# Supplementary material for: Metabolite profiling and transcriptome analyses reveal novel regulatory mechanisms of melatonin biosynthesis in hickory
Source: Hortic Res. 2021 Sep 1;8:196. doi: 10.1038/s41438-021-00631-x (PMC8408178; doi:10.1038/s41438-021-00631-x)
Supplement: Supplementary file 7 — Fig. S4 qRT-PCR analysis, phylogenetic analysis and alignment of the amino-acid sequence of CcAZF2 [file 41438_2021_631_MOESM7_ESM.docx]

**Fig. S4** qRT-PCR analysis, phylogenetic analysis and alignment of the amino acid sequence of CcAZF2. (A) CcAZF2 expression detected by qRT-PCR in hickory treated with 50 mg/L, 100 mg/L, 150 mg/L ABA and water. Three biological replicates from independent RNA extractions for each group of fruit were analyzed. (B) Phylogenetic analysis of CcAZF2 protein from different species. CcAZF2 is boxed. The species information is indicated in parentheses. The information of species follows the GenBank IDs. (C) Alignment of the amino acid sequence of CcAZF2 with NP_174094.1. The conserved Zinc finger C_2_H_2_ type domain is indicated.
