# Supplementary material for: Metabolite profiling and transcriptome analyses reveal novel regulatory mechanisms of melatonin biosynthesis in hickory
Source: Hortic Res. 2021 Sep 1;8:196. doi: 10.1038/s41438-021-00631-x (PMC8408178; doi:10.1038/s41438-021-00631-x)
Supplement: Supplementary file 8 — Fig. S5 qRT-PCR analysis, phylogenetic analysis and alignment of the amino-acid sequence of CcEIN3-1, CcEIN3-2 [file 41438_2021_631_MOESM8_ESM.docx]

**Fig. S5** qRT-PCR analysis, phylogenetic analysis and alignment of the amino acid sequence of CcEIN3-1, CcEIN3-2. (A) and (B) CcEIN3-1, CcEIN3-2 expression detected by qRT-PCR in hickory treated with 500 mg/L, 1000 mg/L, 1500 mg/L Ethrel and water. Three biological replicates from independent RNA extractions for each group of fruit were analyzed. (C) and (D) Phylogenetic analysis of the protein of CcEIN3-1, CcEIN3-2 from different species. CcEIN3-1, CcEIN3-2 are boxed. The species information is indicated in parentheses. The species information follow the GenBank IDs. (C) Alignment of the amino acid sequence of CcEIN3-1 and CcEIN3-2 with NP_188713.1. The conserved Heme-binding NEAT domain is indicated.
