# Supplementary material for: Metabolite profiling and transcriptome analyses reveal novel regulatory mechanisms of melatonin biosynthesis in hickory
Source: Hortic Res. 2021 Sep 1;8:196. doi: 10.1038/s41438-021-00631-x (PMC8408178; doi:10.1038/s41438-021-00631-x)
Supplement: Supplementary file 9 — Fig. S6 qRT-PCR analysis of expression patterns of hormone response genes and melatonin synthesis genes under different hormone treatment with different concentrations and time [file 41438_2021_631_MOESM9_ESM.docx]

**Fig. S6** qRT-PCR analysis of expression patterns of hormone response genes and melatonin synthesis genes under different hormone treatment with different concentrations and time. (A) The relative expression of *CcEIN2-1* (CCA0749S0005), *CcEIN2-2* (CCA1013S0015), *CcEIN3-1* (CCA1573S0039), *CcEIN3-2* (CCA0578S0087), *CcEIN3-3* (CCA0149S0002), *CcEIN3-4* (CCA0892S0010), *CcEIN3-6* (CCA0613S0076), *CcEIN3-7* (CCA0892S0010) and *CcEIN4-1* (CCA0627S0018) in hickory treated with 500 mg/L, 1000 mg/L, 1500 mg/L Ethrel and water for three days, and the expression of *CcAZF2* (CCA0859S0013) and *CcAZF3* (CCA1479S0014) in hickory treated with 50 mg/L, 100 mg/L, 150 mg/L ABA and water for three days. (B) The expression pattern of the same genes as in (A) after six days of treatment. (C) The relative expression of *CcTDC1*, *CcT5H1*, *CcSNAT1*, *CcCOMT1*, and *CcASMT1* in hickory treated with 500 mg/L, 1000 mg/L, 1500 mg/L Ethrel and water or treated with 50 mg/L, 100 mg/L, 150 mg/L ABA and water for three days. (D) The relative expression of *CcTDC1*, *CcT5H1*, *CcSNAT1*, *CcCOMT1*, and *CcASMT1* in hickory treated with 500 mg/L, 1000 mg/L, 1500 mg/L Ethrel and water or treated with 50 mg/L, 100 mg/L, 150 mg/L ABA and water for six days. Three biological replicates from independent RNA extractions for each group of fruit were analyzed.
